# Knowledge, attitude, and practices of nurses during the late stage of COVID-19 in Turkey: cross-sectional study

**DOI:** 10.1590/1980-220X-REEUSP-2024-0400en

**Published:** 2025-07-25

**Authors:** Tuğba Yardımcı Gürel, Özlem Güner

**Affiliations:** 1 Sinop University, Faculty of Health Sciences, Sinop, Turkey.

**Keywords:** Attitude, COVID-19, Knowledge, Nurses, Atitude, COVID-19, Conhecimento, Enfermeiros

## Abstract

**Objective:**

To investigate nurses’ knowledge, attitudes, and practices (KAP) regarding COVID-19 during its late phase.

**Method:**

A descriptive and cross-sectional study was conducted on 354 nurses. The Demographic Information Form and the Knowledge, Attitude, and Practices Scale for COVID-19 (KAP COVID-19) were used to collect data. Data were evaluated using percentages, averages, one-way ANOVA, T-tests, Pearson correlation tests and regression analysis, with significance p < 0.05.

**Results:**

The mean COVID-19 knowledge score of the participants was 7.64 ± 1.54. Nurses had a moderate level of knowledge, a positive attitude towards controlling the pandemic, and a positive attitude towards Turkey’s ability to win the battle against this virus. A discernable relaxation was observed in their COVID-19-related practices, such as wearing masks and avoiding crowded places. It was found that female gender and higher education level were influential in increasing COVID-19 knowledge scores (p < 0.05).

**Conclusion:**

Identifying factors influencing healthcare providers’ attitudes and behaviors towards the virus can guide the development of targeted training programs and policies during the pandemic.

## RESUMO

**Objetivo:**

Investigar o conhecimento, as atitudes e as práticas (CAP) dos enfermeiros em relação à COVID-19 durante sua fase tardia.

**Método:**

Estudo descritivo e transversal realizado com 354 enfermeiros. Utilizaram-se o Formulário de Informações Demográficas e a Escala de Conhecimento, Atitude e Práticas sobre a COVID-19 (CAP COVID-19) para coleta de dados. Os dados foram analisados por meio de porcentagens, médias, ANOVA unidirecional, testes t, correlação de Pearson e análise de regressão, com significância de p < 0.05.

**Resultados:**

A média de pontuação de conhecimento sobre a COVID-19 foi de 7.64 ± 1.54. Os enfermeiros apresentaram conhecimento moderado, atitude positiva quanto ao controle da pandemia e confiança na capacidade da Turquia de vencer a luta contra o vírus. Observou-se um relaxamento nas práticas relacionadas à COVID-19, como o uso de máscaras e a evitação de locais com aglomeração. O sexo feminino e o maior nível de escolaridade influenciaram positivamente o aumento das pontuações de conhecimento (p < 0.05).

**Conclusão:**

A identificação dos fatores que influenciam as atitudes e comportamentos dos profissionais de saúde em relação ao vírus pode orientar o desenvolvimento de programas de treinamento e políticas direcionadas durante a pandemia.

## INTRODUCTION

Four years after the emergence of coronavirus disease 2019 (COVID-19), the world continues to grapple with the novel COVID-19. As of July 2022, approximately 567 million confirmed cases and approximately 6.3 million deaths have been reported worldwide^(1)^. The first COVID-19 case in Turkey was announced by the Ministry of Health on March 11, 2020, initiating pandemic response efforts^(2)^. The Republic of Turkey implemented measures in the fight against the pandemic, including restrictions on international flights, mandatory mask-wearing, and curfews. As a result of the implementation of measures and vaccination campaigns, a gradual normalization process began in May 2021 due to the decrease in the number of cases. In May 2022, the mandatory mask requirement was abolished in all areas outside healthcare facilities^(3)^. The COVID-19 pandemic has imposed a substantial strain on the healthcare system, exerting pressure on healthcare workers, among others^(4)^. As of January 2023, 2,087 healthcare workers have lost their lives due to the pandemic^(5)^. Healthcare workers have a high likelihood of becoming infected themselves due to frequent contact with infected individuals^(6)^. The World Health Organization (WHO), therefore, recommends preventing transmission by protecting healthcare workers and individuals in close contact with^(7)^. Healthcare workers also play a crucial role in controlling the spread of the pandemic. Nurses, who actively contribute to the protection and promotion of health, have taken an important role in the fight against COVID-19 by being at the forefront and risking their lives^(8,9)^. With the rapid increase in the number of cases, a significant number of healthcare workers worldwide lost their lives^(10)^. It is noted that the spread of the disease among healthcare workers is associated with overcrowding, lack of isolation facilities, and contact with contaminated environments. Additionally, it is believed that some healthcare workers may have insufficient knowledge and awareness regarding infection control practices, which could contribute to increased rates of infection^(6,11)^. It is emphasized that inadequate knowledge and incorrect attitudes among healthcare workers can directly affect practices, leading to delayed diagnosis, weakened infection control measures, and the spread of the disease. Knowledge, Attitude, and Practice (KAP) studies aim to identify gaps in knowledge and behavioral patterns among various interest groups and to implement interventions that enhance the quality of health services provided to patients. Therefore, it is believed that understanding the KAP of nurses, who are healthcare providers, regarding infection risk, along with potential risk factors, may help predict the late-term outcomes of COVID-19. In this context, this study provides valuable insights into how nurses’ knowledge, attitudes, and practices evolve during the late phase of a pandemic, highlighting key factors that influence their behaviors after the acute crisis period has passed. Understanding these changes can help healthcare policymakers and institutions develop targeted interventions to maintain adherence to infection control measures and prevent complacency in future health crises. The aim of the present study was to investigate nurses’ knowledge, attitudes, and practices regarding COVID-19 during its late phase.

## METHODS

### Study Design and Sample

This descriptive and cross-sectional study was conducted with nurses working at Sinop Atatürk State Hospital. Sample selection was not performed and the aim was to reach the entire population. Nurses who agreed to participate were included in the study. The inclusion criteria were: (1) being a registered nurse actively working in the hospital during the study period, and (2) voluntary participation. The exclusion criteria included: (1) nurses on extended leave or maternity leave, and (2) those who did not complete the questionnaire fully. The study was completed with 354 nurses who met the research criteria. Study sample corresponded to 91.7% of the population (N = 386). Data were collected online between May 2022 and July 2022 via Google Forms. This study was reported in accordance with the Strengthening the Reporting of Observational Studies in Epidemiology (STROBE) checklist for cross-sectional studies.

### Data Collection

The Demographic Information Form and the Knowledge, Attitude, and Practices Scale for COVID-19 (KAP COVID-19) were used to collect data. Data collection took place over a period of eight weeks, from May to July 2022. Participants were informed about the study via email and social media platforms to encourage participation. To minimize potential biases, we ensured anonymity in data collection to reduce social desirability bias. Additionally, participants were informed that there were no right or wrong answers, encouraging them to provide honest responses. The online nature of data collection may have introduced selection bias, as participation required internet access.

*Demographic information form*: this form was developed by the researchers to collect demographic information about nurses. The form consists of a total of 27 questions, including age, gender, marital status, education level, work experience, duration of employment in the current clinic, weekly working hours, and information related to physical (sleep, exercise, healthy eating, etc.) and mental (stress, anxiety, etc.) well-being.

*Knowledge, attitude, and practices scale for COVID-19 (KAP COVID-19)*: This form was adapted into Turkish by Kurt et al.^(12)^ from the version developed by Zhong et al.^(13)^, comprises a total of 16 items and 5 factors. The first 12 items of the scale are aimed at measuring the level of knowledge and consist of three factors: Clinical Presentations (Items 1, 2, 3, 4), Transmission Routes (Items 5, 6, 7), and Prevention and Control (Items 8, 9, 10, 11, 12). The scale consists of options “true,” “false,” and “don’t know.” 1 point is as-signed for correct answers, while 0 points are assigned for incorrect answers or when “don’t know” is selected. The total scale score can range from a minimum of 0 to a maximum of 12. High scores indicate a high level of knowledge about COVID-19, while low scores indicate a low level of knowledge. Attitudes towards COVID-19 are measured by two items regarding consensus on the ultimate control of COVID-19 and confidence in winning the battle against COVID-19. Practices are evaluated by two items, which inquire about going to a crowded place recently and wearing a mask when going out. The Cronbach’s Alpha value was found to be 0.74 for the overall scale^(12)^.

### Data Analysis

Data analysis was conducted using the SPSS version 25.0 software program. The data were presented as numbers, percentages, and means. Parametric tests were used for statistical analysis as the data were normally distributed. To provide a more precise estimation of population parameters, 95% confidence intervals (CIs) were calculated for the main scale dimensions. These confidence intervals provide additional insight into the dispersion and reliability of the data. Knowledge scores, attitudes, and practices were evaluated with respect to demographic characteristics using independent samples t-test, one-way ANOVA, or Chi-square test as appropriate. The relationship between variables was tested using Pearson correlation analysis. Simple linear regression analysis was used to determine the variables predicting the level of knowledge about COVID-19. The internal consistency of the KAP COVID-19 scale was assessed using Cronbach’s Alpha, which was found to be 0.72 in the present study. A p value of <0.05 was considered statistically significant in all analyses.

### Ethical Aspects

The study received approval from the Sinop University Institutional Review Board for Human Research (Approval No: 2022/31, dated March 24, 2022). Additionally, participants were provided with information about the purpose of the study at the beginning of the online form, and access to the forms was granted after they checked the box indicating their willingness to participate in the study. None of the authors had access to the respondents’ personal identifying information that could identify individual participants during or after data collection.

## RESULTS

The study was conducted with 354 nurses. The mean age of the participants was 33.04 ± 8.55 years (ranging from 21 to 59), and the average work experience was 10.83 ± 9.16 years (ranging from 1 to 39). It was determined that 75.1% of the participants were female, 52.5% were married, 70.3% were university graduates, and 71.2% had a moderate-income level. It was found that 85.6% of the participants did not have any chronic illnesses, 47.7% worked during regular hours and had night shifts, and 59.3% worked between 41–50 hours per week (Table 1).

**Table 1:** Comparison of participants’ sociodemographic characteristics and KAP COVID-19 scores – Sinop, Turkey, 2022.

	N	%	Clinical presentations	Transmission routes	Prevention and control	Total
**Age**						
20–29	168	47.5	2.88 ± 0.81	1.10 ± 0.59	3.58 ± 0.85	7.56 ± 1.53
30–39	96	27.1	2.95 ± 0.91	1.14 ± 0.58	3.56 ± 0.89	7.66 ± 1.46
40–49	77	21.8	3.09 ± 0.83	1.18 ± 0.7	3.46 ± 1.10	7.74 ± 1.69
50–59	13	3.7	3.07 ± 1.03	1.23 ± 0.83	3.53 ± 0.77	7.84 ± 1.34
**Test statistic**			F = 1.162 p = 0.324	F = 0.418 p = 0.740	F = 0.286 p = 0.836	F = 0.325 p = 0.807
**Gender**						
Female	266	75.1	3.03 ± 0.77	1.09 ± 0.56	3.58 ± 0.84	7.71 ± 1.32
Male	88	24.9	2.82 ± 0.9	1.29 ± 0.76	3.62 ± 0.84	7.74 ± 1.53
**Test statistic**			t = 2.121 p = 0.035*	t = −2.590 p = 0.010*	t = −0.347 p = 0.729	t = −0.125 p = 0.901
**Marital status**						
Married	186	52.5	3.02 ± 0.83	1.16 ± 0.63	3,56 ± 0,83	7,75 ± 1,24
Unmarried	168	47.5	2.94 ± 0.79	1.12 ± 0.61	3,62 ± 0,84	7,69 ± 1,51
**Test statistic**			t = 0.549 p = 0.538	t = 0.644 p = 0.520	t = −0.864 p = 0.388	t = 0.050 p = 0.960
**Education**						
Health vocational high school	38	10.7	2.34 ± 1.09	1.42 ± 0.94	3.31 ± 1.43	7.07 ± 2.69
Associate’s degree	42	11.9	3.04 ± 0.76	0.97 ± 0.60	3.47 ± 1.01	7.50 ± 1.41
Bachelor’s degree	249	70.3	3.01 ± 0.79	1.12 ± 0.55	3.59 ± 0.81	7.72 ± 1.31
Master degree or higher	25	7.1	3.16 ± 0.80	1.12 ± 0.60	3.60 ± 0.70	7.88 ± 1.30
**Test statistic**			F = 7.993 p = 0.000*	F = 3.700 p = 0.012*	F = 1.134 p = 0.335	F = 2.288 p = 0.078
**Income**						
Less than expenses	70	19.8	3.05 ± 0.79	1.21 ± 0.67	3.68 ± 0.87	7.95 ± 1.31
Equal to expenses	252	71.2	2.93 ± 0.88	1.13 ± 0.61	3.50 ± 0.95	7.58 ± 1.62
More than expenses	32	9.0	2.87 ± 0.75	0.93 ± 0.50	3.59 ± 0.66	7.40 ± 1.18
**Test statistic**			F = 0.699 p = 0.498	F = 2.187 p = 0.114	F = 1.066 p = 0.346	F = 2.035 p = 0.132
**Tobacco use**						
Yes	142	40.1	2.84 ± 0.93	1.23 ± 0.72	3.59 ± 1.03	7.66 ± 1.81
No	212	59.9	3.02 ± 0.79	1.07 ± 0.54	3.52 ± 0.82	7.62 ± 1.33
**Test statistic**			t = −1.987 p = 0.048*	t = 2.405 p = 0.017*	t = 0.682 p = 0.496	t = 0.277 p = 0.782
**Alcohol use**						
Yes	65	18.4	2.58 ± 1.05	1.21 ± 0.85	3.21 ± 1.30	7.01 ± 2.34
No	289	81.6	3.03 ± 0.77	1.11 ± 0.55	3.62 ± 0.78	7.78 ± 1.25
**Test statistic**			t = −3.948 p = 0.000*	t = 1.141 p = 0.255	t = −3.306 p = 0.001*	t = −3690 p = 0.000*
**Chronic Disease Status**						
Yes	51	14.4	3.13 ± 0.74	1.09 ± 0.60	3.58 ± 0.87	7.82 ± 1.60
No	303	85.6	2.92 ± 0.86	1.14 ± 0.62	3.54 ± 0.92	7.61 ± 1.52
**Test statistic**			t = 1.654 p = 0.099	t = −0.464 p = 0.643	t = 0.314 p = 0.754	t = 0.913 p = 0.362
**Work experience (years)**						
0–9	190	53.7	2.87 ± 0.81	1.10 ± 0.58	3.61 ± 0.83	7.59 ± 1.47
10–19	91	25.7	3.00 ± 0.86	1.21 ± 0.66	3.53 ± 0.89	7.75 ± 1.50
20–29	62	17.5	3.11 ± 0.87	1.11 ± 0.68	3.33 ± 1.172	7.56 ± 1.78
30–39	11	3.1	3.00 ± 1.18	1.18 ± 0.60	3.72 ± 0.78	7.90 ± 1.51
**Test statistic**			F = 1.307 p = 0.272	F = 0.804 p = 0.492	F = 1.564 p = 0.197	F = 0.393 p = 0.758
**Department**						
Emergency room	74	20.9	2.82 ± 0.88	1.16 ± 0.74	3.56 ± 0.93	7.55 ± 1.67
Surgical departments	57	16.1	3.03 ± 0.80	1.05 ± 0.39	3.66 ± 0.78	7.75 ± 1.15
Internal departments	73	20.6	2.73 ± 0.97	1.10 ± 0.51	3.39 ± 1.12	7.24 ± 1.80
Intensive care unit	38	10.7	2.97 ± 0.67	1.07 ± 0.48	3.55 ± 0.86	7.60 ± 1.15
Other (Polyclinic etc.)	112	31.6	3.13 ± 0.79	1.19 ± 0.73	3.58 ± 0.83	7.91 ± 1.51
**Test statistic**			F = 3.021 p = 0.018*	F = 0.659 p = 0.621	F = 0.770 p = 0.545	F = 3.227 p = 0.046*
**Work schedule**						
In shifts	102	28.8	2.68 ± 0.91	1.01 ± 0.54	3.37 ± 1.12	7.07 ± 1.90
Daytime only	83	23.4	3.12 ± 0.86	1.16 ± 0.64	3.65 ± 0.73	7.93 ± 1.05
Day ± Duty	169	47.7	3.03 ± 0.77	1.18 ± 0.65	3.60 ± 0.84	7.83 ± 1.40
**Test statistic**			F = 7.641 p = 0.001*	F = 3.527 P = 0.044*	F = 2.785 p = 0.063	F = 10.202 p = 0.000*
**Weekly working hours**						
40 hours or less	65	18.4	2.93 ± 1.02	1.07 ± 0.62	3.38 ± 1.08	7.40 ± 1.87
41–50 hours	210	59.3	2.89 ± 0.83	1.13 ± 0.62	3.58 ± 0.84	7.61 ± 1.47
51 hours or more	79	22.3	3.12 ± 0.72	1.17 ± 0.63	3.59 ± 0.94	7.89 ± 1.38
**Test statistic**			F = 2.136 p = 0.120	F = 0.463 p = 0.630	F = 1.310 p = 0.271	F = 1.933 p = 0.146

*p value < 0.05 is considered statistically significant.

The mean COVID-19 Knowledge score of the participants was 7.64 ± 1.54 (95% CI: 7.48 – 7.80). The mean clinical presentations score was 2.95 ± 0.85 (95% CI: 2.86 – 3.04). The mean transmission routes score was 1.14 ± 0.62 (95% CI: 1.07 – 1.20). The mean prevention and control score was 3.55 ± 0.91 (95% CI: 3.45 – 3.65). The mean scores of KAP COVID-19 scale and its sub dimensions according to the sociodemographic characteristics of the participants are presented in Table 1. Statistically significant differences were found in the clinical presentations sub-dimension scores with respect to gender, education level, smoking and alcohol use, department, and work schedule (p < 0.05). Statistically significant higher mean scores in the clinical presentations sub-dimension scores were observed among participants who were female, had post-graduate degrees, were non-smokers and non-drinkers, worked exclusively day shifts, and were employed in other units such as outpatient clinics and surgical units. Statistically significant differences were observed in the transmission routes sub-dimension scores with respect to gender, education level, smoking status, and work schedule (p < 0.05). It was determined that female nurses, nurses with an associate’s degree, non-smokers, and nurses working in shifts had significantly lower transmission routes scores. A statistically significant difference was found in the prevention and control sub-dimension scores with respect to alcohol use, with non-drinkers having significantly higher mean scores (p < 0.05). Statistically significant differences were found in COVID-19 Knowledge scores with respect to alcohol use, department, and work schedule (p<0.05). Non-drinkers, those working exclusively day shifts, as well as those working in other units such as outpatient clinics and surgical units, were found to have significantly higher COVID-19 Knowledge scores (Table 1).

Participants’ characteristics regarding COVID-19 are presented in Table 2. It was determined that 60.7% of the participants had been infected with COVID-19, 37.9% had a family member who had been infected, and 79.4% had provided care to a COVID-19 patient. There was no statistically significant difference in COVID-19 Knowledge scores among participants, regardless of whether they themselves had been infected, whether their family members had been infected, or whether they had provided care to an infected patient (p > 0.05). Statistically significant differences were found in clinical presentation sub-dimension scores regarding the infection status of relatives (p < 0.05). Similarly, statistically significant differences were found in prevention and control sub-dimension scores and COVID-19 Knowledge scores regarding contact with infected patients (p < 0.05). It was determined that participants who rarely or never had contact with infected patients had significantly higher COVID-19 Knowledge scores and prevention and control sub-dimension scores (Table 2).

**Table 2:** Comparison of KAP COVID-19 scores with respect to other characteristics – Sinop, Turkey, 2022.

	N	%	Clinical presentations	Transmission routes	Prevention and control	Total
**Infected with COVID-19**						
Yes	215	60.7	3.01 ± 0.82	1.09 ± 0.56	3.58 ± 0.86	7.69 ± 1.43
No	139	39.3	2.85 ± 0.89	1.19 ± 0.70	3.50 ± 1.00	7.55 ± 1.68
**Test statistic**			t = 1.754 p = 0.080	t = −1.362 p = 0.175	t = 0.753 p = 0.452	t = 0.857 p = 0.392
**Infection of a family member**						
Yes	134	37.9	2.88 ± 0.87	1.14 ± 0.54	3.62 ± 0.86	7.65 ± 1.48
No	220	62.1	3.00 ± 0.83	1.12 ± 0.67	3.50 ± 0.94	7.63 ± 1.57
Test statistic			t = −1.277 p = 0.202	t = 0.321 p = 0.748	t = 1.217 p = 0.225	t = 0.147 p = 0.883
**Infection of a relative**						
Yes	117	33.1	2.78 ± 0.80	1.10 ± 0.57	3.53 ± 0.93	7.42 ± 1.51
No	237	66.9	3.03 ± 0.86	1.15 ± 0.64	3.55 ± 0.91	7.74 ± 1.54
**Test statistic**			t = −2.631 p = 0.009*	t = −0.699 p = 0.485	t = −0.178 p = 0.859	t = −1.842 p = 0.066
**Caring for COVID-19 infected patient**						
Yes	281	79.4	2.96 ± 0.89	1.12 ± 0.61	3.54 ± 0.93	7.62 ± 1.57
No	73	20.6	2.93 ± 0.69	1.17 ± 0.67	3.57 ± 0.84	7.68 ± 1.42
**Test statistic**			t = −0.699 p = 0.485	t = −0.699 p = 0.485	t = −0.699 p = 0.485	t = −0.699 p = 0.485
**Contact with infected patient**						
Always	129	36.4	3.00 ± 0.83	1.18 ± 0.69	3.64 ± 0.83	7.83 ± 1.44
Sometimes	155	43.8	2.87 ± 0.92	1.07 ± 0.55	3.40 ± 1.05	7.34 ± 1.67
Rarely/Never	70	19.8	3.04 ± 0.71	1.17 ± 0.63	3.71 ± 0.66	7.92 ± 1.28
**Test statistic**			F = 1.371 p = 0.255	F = 1.212 p = 0.299	F = 3.920 p = 0.021*	F = 5.184 p = 0.006*

* p value < 0.05 is considered statistically significant.

The relationship between the participants’ perceived emotional and physical health levels during the pandemic and KAP COVID-19 scores is shown in Table 3. A statistically significant, weak positive correlation was found between COVID-19 Knowledge scores and feeling anxious, stressed, depressed and happy during the pandemic (p < 0.05). As the level of knowledge about COVID-19 increases, the level of feeling anxious, stressed, de-pressed, and happy during the pandemic also increases. A statistically significant, weak positive correlation was observed between the clinical presentations score and feeling anxious, stressed and happy during the pandemic. Conversely, a statistically significant, weak negative correlation was observed between the clinical presentations score and the level of healthy eating. As clinical presentations score increases, the level of feeling anxious, stressed and depressed during the pandemic increases and the level of healthy eating decreases. There was a statistically significant, weak positive correlation between transmission routes scores and the level of fatigue. As transmission routes score increases, the level of fatigue also increases. A statistically significant, weak positive correlation was found between prevention and control scores and feeling happy during the pandemic. As prevention and control score increases, the level of feeling happy during the pandemic also increases (Table 3).

**Table 3:** Relationship between participants’ perceived emotional and physical health during the pandemic and kap covid-19 scores – Sinop, Turkey, 2022.

	M ± SD	Min–Max	Clinical presentations	Transmission routes	Prevention and control	Total
Being satisfied with work life	5.04 ± 2.32	0–10	0.052	−0.048	0.038	0.68
Feeling anxious during the pandemic	7.22 ± 2.56	0–10	0.117*	0.084	0.006	0.115*
Feeling stressed during the pandemic	7.23 ± 2.55	0–10	0.152**	0.067	0.032	0.159**
Feeling depressed during the pandemic	6.75 ± 2.63	0–10	0.090	0.078	0.052	0.135*
Level of happiness during the pandemic	6.71 ± 2.38	0–10	0.182**	0.068	0.126*	0.208**
Sleep level	5.08 ± 2.39	0–10	−0.062	−0.007	0.044	−0.007
Fatigue level	7.36 ± 2.13	0–10	−0.003	0.144**	0.052	0.086
Physical activity level	6.58 ± 2.31	0–10	−0.063	0.024	0.073	0.028
Healthy nutrition level	6.23 ± 2.08	0–10	−0.128*	0.025	0.004	−0.085

Simple linear regression analysis was used to determine the variables predicting the level of knowledge about COVID-19 (Table 4). The analysis revealed that the level of feeling happy during the pandemic, working only day shifts, not consuming alcohol, and not having contact with an infected patient, explained 10% of the variance in participants’ level of knowledge about COVID-19 (p < 0.001).

**Table 4:** Linear regression analysis results for predicting the level of knowledge about COVID-19 – Sinop, Turkey, 2022.

Variables	B	S.H.	β	t	p	VIF
Constant	6.295	.328		19.183	.000	1.009
Level of happiness during the pandemic	.144	.033	.224	4.386	.000	1.023
Work Schedule	.240	.186	.066	1.286	.199	1.015
Alcohol use	.695	.203	.175	3.419	.001	1.018
Contact with infected patient	−.307	.198	−.080	−1.553	.121	1.009
R = .316 R^2^ = .100 F = 9.662 p < 0.001						

**Figure 1:**
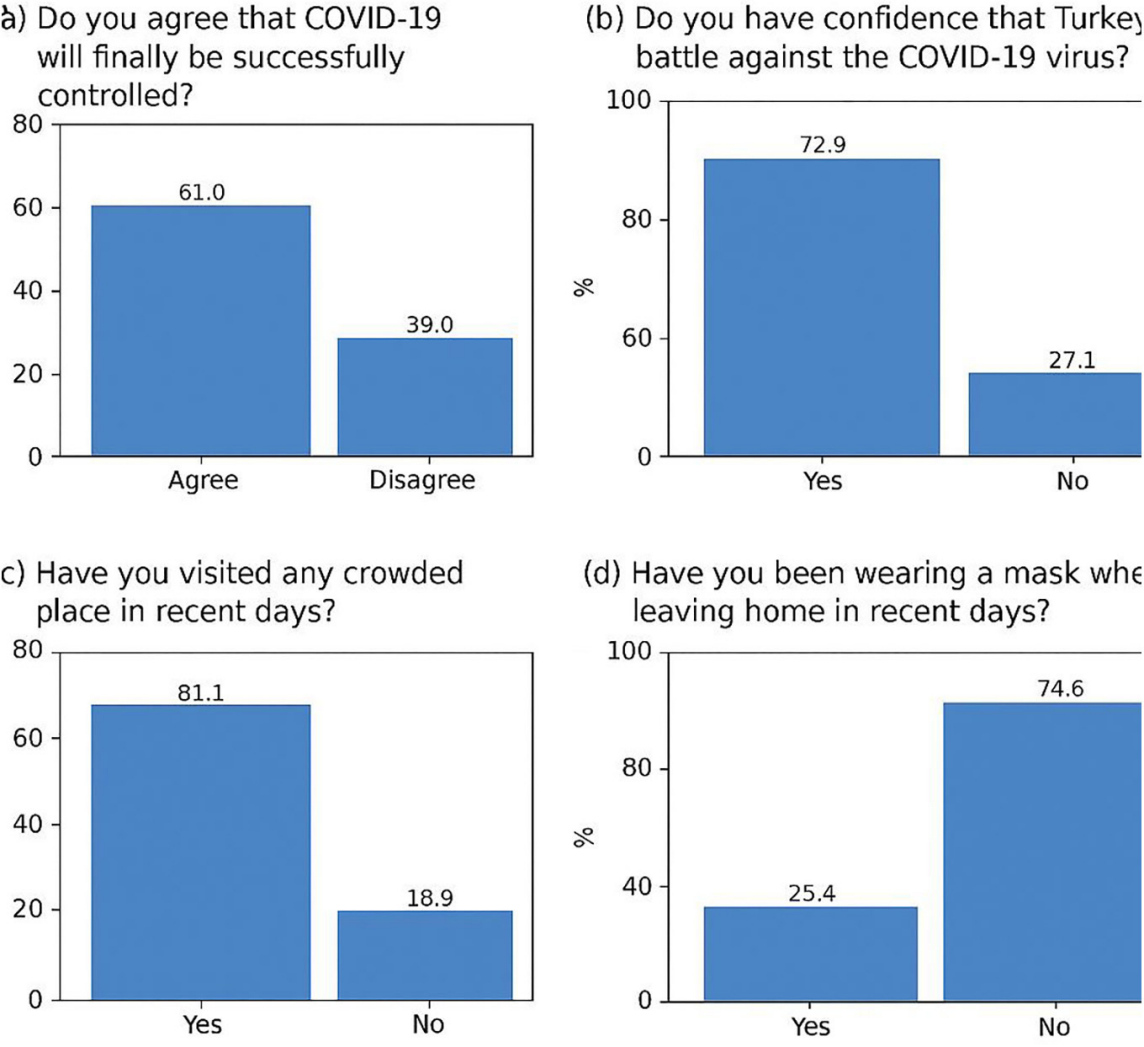
Attitudes and practices towards COVID-19.

When the attitude sub-dimension of the scale was analyzed, it was found that 61% (216) of the participants answered “Agree” to the question “Do you agree that COVID-19 will finally be successfully controlled?”, and 72.9% (258) of the participants answered “Yes” to the question “Do you have confidence that Turkey can win the battle against the COVID-19 virus?”. When the practices sub-dimension of the scale was analyzed, it was found that 81.1% (287) of the participants answered “Yes” to the question “Have you visited any crowded place in recent days?”, and 74.6% (264) answered “No” to the question “Have you been wearing a mask when leaving home in recent days?” (Figure 1).

## DISCUSSION

Prevention is the only solution to a disease where curative treatment is not available. As long as the majority of the population is not vaccinated and the country reaches the required herd immunity level, preventive measures are largely dependent on the KAP of the affected population. Healthcare workers’ knowledge and behavior are crucial to prevent the spread of infection. If neglected, healthcare workers not only risk becoming potential vectors themselves but also jeopardize their own lives^(14)^. This study was conducted during the late period of COVID-19 in Turkey to investigate KAP in relation to the management of COVID-19 among nurses in the workplace and in their personal lives. To the best of our knowledge, this study is the first comprehensive assessment of knowledge, attitudes, and practices among frontline healthcare workers, particularly nurses, regarding the late phase of COVID-19 in Turkey. In this predominantly female, well-educated population, the correct response rate in the knowledge questionnaire was 63.67%, with an average knowledge score of 7.64 ± 1.54. These results indicate that the majority of respondents had a moderate knowledge of COVID-19. The knowledge score obtained among health workers in the present study was lower compared to other international studies conducted in Nigeria (92%), China (89%), and Saudi Arabia (84%)^(11,15,16)^. Other studies conducted in Turkey found that the level of knowledge of healthcare workers about COVID-19 was 92% and 94%, respectively, which were higher compared to the present study^(17,18)^. Similarly, Ejeh et al.^(19)^ found that 88% of healthcare workers answered the questions correctly, with a mean knowledge score of 7.1. These studies were carried out during the early stages of the pandemic when limited information was available. In contrast, the present study was conducted during a later phase, when there were abundant information available, and personal experiences also shaped opinions. This difference may have led to differences in the findings. There are also results consistent with the present study in the literature^(20,21,22,23)^. In addition to this information, the present study revealed that the majority of nurses had sufficient knowledge about the clinical symptoms and signs of the disease (73.75%) as well as its prevention and control measures (71%). However, their knowledge regarding the modes of transmission (37.67%) was found to be inadequate. These findings are consistent with studies from Vietnam and India, which suggest that healthcare workers have sufficient knowledge about the transmission and clinical symptoms of COVID-19^(21,24)^. Similarly, Bangani et al.^(22)^ found moderately good (78%), low (55%), and moderate (65%) knowledge scores of intensive care nurses for COVID-19, attitude scores, and practice scores^(22)^. In this study conducted during the mid-phase of COVID-19, it was noted that the particularly low attitude scores were influenced by limited infection prevention and control training, insufficient time for implementation, and a lack of personal protective equipment. The low scores in the present study may be due to the emergence of a plethora of new information regarding the spread of the disease and transmission routes during the study period, coupled with inadequate training provided to nurses on this new information. Additionally, the inability of nurses to keep up with current information due to their demanding work schedules may have contributed to this situation. The analyses conducted in the present study revealed that overall, female nurses with a bachelor’s degree or higher education level, who did not use alcohol or tobacco, and worked only day shifts in outpatient units, tended to have higher knowledge scores. Having a higher level of education is one of the factors contributing to having more experience due to increased access to information and knowledge acquisition opportunities. In the present study, it was found that nurses with a bachelor’s degree or higher education level had higher levels of COVID-19 knowledge compared to those with lower levels of education, which is consistent with a previous study conducted in Turkey^(17)^. In a similar study conducted in Saudi Arabia, Almohammed et al.^(20)^ reported that female gender and higher education level positively affected knowledge scores. This finding is supported by international studies conducted in Yemen, Ethiopia, and Nigeria^(15,25,26)^. A possible reason for this could be that healthcare providers with higher levels of education may have better opportunities to access local and international knowledge, research and training platforms than their colleagues^(26)^. There are also studies where, contrary to our findings, no significant differences were found between socio-demographic characteristics and knowledge levels^(16,19)^.

In the present study, we investigated the relationship between participants’ self-reported emotional and physical health status and their level of knowledge about COVID-19. It was found that during the pandemic, the levels of anxiety, stress, and depression also increased as knowledge level increased. As stated in a previous study, considering the serious impact of the pandemic on society, it is expected that there would be a general presence of fear and anxiety in the community; this can also affect people’s mental health and societal behaviors^(27)^. Alrubaiee et al.^(25)^ found a significant positive relationship between knowledge level and anxiety, similar to the present study. It was noted that anxiety levels among healthcare workers could be attributed to concerns about becoming infected, particularly considering the uncontrollable nature of the out-break and factors such as shortages of healthcare facilities and personal protective equipment. In the present study, we found a positive correlation between the level of knowledge regarding the clinical symptoms of COVID-19 and feeling anxious and stressed during the pandemic period. Conversely, a negative correlation was found between the level of healthy eating and these feelings. The similarity between the symptoms and signs of the disease and classic upper respiratory tract infection symptoms, along with specific symptoms of the disease, may have caused participants to become concerned and experience negative emotional feelings when they or their loved ones exhibited these symptoms. A decrease in healthy eating can be attributed to deterioration of eating habits as a result of anxiety. Contrary to the other findings, it was found that happiness levels of some participants increased as their level of knowledge increased. This phenomenon may stem from an increase in self-confidence among workers as their level of knowledge rises, leading them to feel safer against the transmission of the virus. There was a significant positive correlation between the level of knowledge about transmission routes and fatigue. The knowledge of the ways in which the virus is transmitted could lead nurses to make more efforts to prevent potential transmission, thus increasing their workload and resulting in a more severe fatigue.

In the present study, regression analysis was conducted to determine the predictors of COVID-19 knowledge level. The results showed that feeling happy during the pandemic, working day shifts, abstaining from alcohol use, and avoiding contact with infected patients explained 10% of the variance in participants’ knowledge level (p < 0.001). These findings are important since there are limited studies examining KAP among nurses with respect to various characteristics. Additionally, identifying demographic factors associated with KAP could be beneficial for public health policymakers and healthcare professionals in recognizing target populations for the prevention of COVID-19 and health education initiatives.

The majority of nurses exhibited an optimistic attitude towards the COVID-19 pandemic: 61% believed that COVID-19 would be successfully controlled, and 72.9% believed that Turkey could win the battle against the virus. In a study investigating the knowledge, preventive behaviors, and risk perception of healthcare workers regarding the COVID-19 pandemic in Turkey, it was reported that the vast majority of participants (84%) believed that the outbreak would be controlled^(17)^. These rates, despite being conducted at the early stages of the pandemic, are more optimistic compared to the present study. The results obtained in the present study may be attributed to the fact that the pandemic has been ongoing for approximately three years and has yet to be fully controlled. At the time this study was conducted, the preventive restrictions for COVID-19 implemented in Turkey had been lifted. This may have resulted in the less strict practices observed: The vast majority of participants reported going to crowded places (81.1%) and not wearing masks when leaving their homes (74.6%). This finding aligns with previous studies that have demonstrated a decline in adherence to preventive measures as public risk perception decreased. Petherick et al.^(28)^ found that adherence to protective behaviors diminished globally as restrictions eased and pandemic fatigue emerged. Similarly, Seyd and Bu^(29)^ highlighted that public compliance with COVID-19 measures was strongly influenced by perceived risk and trust in government institutions, with compliance levels decreasing as concerns about the virus lessened. These findings suggest that long-term adherence to protective measures is significantly shaped by both institutional trust and perceived threat levels. In another study conducted during the earlier stages of the pandemic, Arslanca et al.^(17)^ reported that the main preventive behaviors outside of work were the use of masks (96%) and avoiding crowded places (91.63%). Although our study was carried out during a period when restrictions aimed at controlling the pandemic had been abolished, the fact that nearly 20–30% of participants continued to voluntarily adhere to preventive measures is indicative of their ongoing concerns. Similar to our study, in a study conducted by Juttla et al.^(30)^ during the mid-stages of the pandemic in Kenya, the majority of healthcare workers were reported to have a negative attitude towards COVID-19, despite having a high level of preventive practices. It was noted that 66.3% of them avoided patients with symptoms and signs of COVID-19. The authors emphasized that this indicated healthcare workers’ lack of confidence in the effectiveness of preventive protocols, regardless of their adherence to them. The organizational climate can significantly impact nurses’ knowledge, attitudes, and practices by influencing access to training, availability of protective measures, and institutional policies. We hypothesize that a supportive organizational climate, characterized by ongoing education and clear infection control policies, facilitates better adherence to preventive measures among nurses.

There are certain limitations to the present study. One limitation is that the cross-sectional research design may not sufficiently explain the cause-and-effect relationship. Additionally, the study was conducted at a single center, and the experiences of healthcare workers in the private sector were not considered. Therefore, the results cannot be generalized to the entire population. Another limitation is that this study was conducted through an online survey. Responses depended on honesty and were influenced by the ability to recall; therefore, they may be subject to recall bias. However, a notable strength of this study is that all nurses working at a large state hospital in Turkey participated. Furthermore, this is the first study to assess the KAP of nurses with regard to COVID-19 during the late stages of the pandemic. The data obtained can serve as a valuable resource for future research and educational endeavors.

## CONCLUSION

The results obtained in the present study revealed important factors related to KAP among nurses who survived the COVID-19 infection. These results make an important contribution to the literature in terms of reflecting the late-stage findings of the pandemic as the study was conducted at a time when the preventive measures were lifted. In general, it was found that nurses had a moderate level of knowledge, a positive attitude towards the control of the pandemic and Turkey’s ability to win the battle against this virus. However, a discernable relaxation was observed in their COVID-19-related practices, such as wearing masks and avoiding crowded places. In addition, it was found that female gender and higher education level were influential to increase knowledge scores. Identifying factors that influence healthcare providers’ attitudes and behaviors towards the virus can guide the development of targeted training programs and policies during pandemic. These efforts can focus on the protection of healthcare workers and help them avoid occupational exposure. The findings also revealed that nurses who survived COVID-19 still experienced feelings of anxiety, stress, and depression during the pandemic period. On the basis of these findings, it may be recommended to provide psychological support in the workplace by focusing on improving job satisfaction and enhancing the quality of health outcomes among nurses with an emphasis on building resilience, empowerment, and preventing burnout. In addition, hospitals should provide continuous professional development programs to raise awareness among all healthcare workers and to support their emotional well-being during the ongoing pandemic. Considering that the data were collected in 2022, additional analyses could include a longitudinal follow-up study to assess how nurses’ knowledge, attitudes, and practices evolve over time. Comparative studies with more recent datasets could also help determine whether adherence to COVID-19 preventive measures has further declined or improved post-pandemic.

## Data Availability

The data supporting the findings of this study are available from the corresponding author upon reasonable request.
