# Supplementary material for: Knowledge, attitude, and practices of nurses during the late stage of COVID-19 in Turkey: cross-sectional study
Source: Rev Esc Enferm USP. 2025 Jul 25;59:e20240400. doi: 10.1590/1980-220X-REEUSP-2024-0400en (PMC12306191; doi:10.1590/1980-220X-REEUSP-2024-0400en)
Supplement: Translation

# Translation: Conocimientos, actitudes y prácticas de las enfermeras durante la fase tardía del COVID-19 en Turquía: estudio transversal

**DOI:** 10.1590/1980-220X-REEUSP-2024-0400es

**Authors:** Tuğba Yardımcı Gürel, Özlem Güner

**Affiliations:** 1 Sinop University, Faculty of Health Sciences, Sinop, Turkey.

**Keywords:** Actitud, COVID-19, Conocimiento, Enfermeras

## Abstract

**Objetivo:**

Investigar los conocimientos, actitudes y prácticas (CAP) de las enfermeras sobre la COVID-19 durante su fase tardía.

**Método:**

Estudio descriptivo y transversal realizado con 354 enfermeras. Se utilizaron el Formulario de Información Demográfica y la Escala de Conocimientos, Actitudes y Prácticas sobre la COVID-19 (CAP COVID-19) para recopilar los datos. Los datos se analizaron mediante porcentajes, medias, ANOVA unidireccional, pruebas t, correlación de Pearson y análisis de regresión, con significancia de p < 0.05.

**Resultados:**

La puntuación media de conocimientos sobre la COVID-19 fue de 7.64 ± 1.54. Las enfermeras mostraron un nivel moderado de conocimiento, una actitud positiva hacia el control de la pandemia y confianza en la capacidad de Turquía para superar la lucha contra el virus. Se observó una relajación notable en las prácticas relacionadas con la COVID-19, como el uso de mascarillas y la evitación de lugares concurridos. Se determinó que el género femenino y un mayor nivel educativo influyeron positivamente en las puntuaciones de conocimientos (p < 0.05).

**Conclusión:**

Identificar los factores que influyen en las actitudes y comportamientos de los profesionales sanitarios frente al virus puede orientar el desarrollo de programas de formación y políticas específicas durante la pandemia.

## INTRODUCCIÓN

Cuatro años después de la aparición de la enfermedad por coronavirus 2019 (COVID-19), el mundo sigue lidiando con el nuevo COVID-19. Hasta julio de 2022, se habían notificado aproximadamente 567 millones de casos confirmados y unos 6,3 millones de muertes en todo el mundo^(1)^. El primer caso de COVID-19 en Turquía fue anunciado por el Ministerio de Sanidad el 11 de marzo de 2020, iniciando los esfuerzos de respuesta a la pandemia^(2)^. La República de Turquía aplicó medidas en la lucha contra la pandemia, incluidas restricciones en los vuelos internacionales, uso obligatorio de mascarillas y toques de queda. Como resultado de la aplicación de las medidas y de las campañas de vacunación, en mayo de 2021 se inició un proceso de normalización gradual debido a la disminución del número de casos. En mayo de 2022 se suprimió la obligatoriedad del uso de mascarilla en todas las zonas fuera de los centros sanitarios^(3)^.

La pandemia de COVID-19 ha impuesto una presión sustancial sobre el sistema sanitario, ejerciendo presión sobre el personal sanitario, entre otros^(4)^. Hasta enero de 2023, 2087 trabajadores sanitarios han perdido la vida a causa de la pandemia^(5)^. El personal sanitario tiene una alta probabilidad de infectarse debido al contacto frecuente con personas infectadas^(6)^. Por ello, la Organización Mundial de la Salud (OMS) recomienda prevenir la transmisión protegiendo a los trabajadores sanitarios y a las personas en estrecho contacto con^(7)^. El personal sanitario también desempeña un papel crucial en el control de la propagación de la pandemia. Las enfermeras, que contribuyen activamente a la protección y promoción de la salud, han asumido un papel importante en la lucha contra el COVID-19 al estar en primera línea y arriesgar sus vidas^(8,9)^. Con el rápido aumento del número de casos, un número significativo de trabajadores sanitarios de todo el mundo perdieron la vida^(10)^. Se señala que la propagación de la enfermedad entre los trabajadores sanitarios está asociada al hacinamiento, la falta de instalaciones de aislamiento y el contacto con entornos contaminados. Además, se cree que algunos trabajadores sanitarios pueden tener conocimientos y concienciación insuficientes sobre las prácticas de control de infecciones, lo que podría contribuir a aumentar las tasas de infección^(6,11)^. Se hace hincapié en que los conocimientos inadecuados y las actitudes incorrectas entre el personal sanitario pueden afectar directamente a las prácticas, lo que conduce a retrasos en el diagnóstico, debilitamiento de las medidas de control de la infección y propagación de la enfermedad.

Los estudios de Conocimientos, Actitudes y Prácticas (CAP) tienen como objetivo identificar las lagunas en los conocimientos y las pautas de comportamiento entre los distintos grupos de interés y aplicar intervenciones que mejoren la calidad de los servicios sanitarios prestados a los pacientes. Por lo tanto, se cree que la comprensión de los CAP de las enfermeras, que son profesionales sanitarios, en relación con el riesgo de infección, junto con los posibles factores de riesgo, puede ayudar a predecir los resultados a largo plazo de la COVID-19. En este contexto, este estudio proporciona valiosas perspectivas sobre cómo evolucionan los conocimientos, actitudes y prácticas de las enfermeras durante la fase tardía de una pandemia, destacando los factores clave que influyen en sus comportamientos una vez pasado el periodo de crisis aguda. Comprender estos cambios puede ayudar a los responsables de las políticas sanitarias y a las instituciones a desarrollar intervenciones específicas para mantener el cumplimiento de las medidas de control de infecciones y evitar la complacencia en futuras crisis sanitarias. El objetivo del presente estudio era investigar los conocimientos, actitudes y prácticas de las enfermeras en relación con la COVID-19 durante su fase tardía.

## MÉTODOS

### Diseño del Estudio y Muestra

Este estudio descriptivo y transversal se realizó con enfermeras que trabajaban en el Hospital Estatal Atatürk de Sinop. No se realizó selección de la muestra y el objetivo era llegar a toda la población. Se incluyeron en el estudio las enfermeras que aceptaron participar. Los criterios de inclusión fueron: (1) ser una enfermera titulada que trabajara activamente en el hospital durante el periodo de estudio, y (2) participación voluntaria. Los criterios de exclusión fueron: (1) las enfermeras con baja prolongada o baja por maternidad, y (2) las que no cumplimentaron el cuestionario en su totalidad. El estudio se completó con 354 enfermeras que cumplían los criterios de investigación. La muestra del estudio correspondía al 91,7% de la población (N = 386). Los datos se recolectaron en línea entre mayo de 2022 y julio de 2022 a través de Google Forms. Este estudio se informó de acuerdo con la lista de verificación Strengthening the Reporting of Observational Studies in Epidemiology (STROBE) para estudios transversales.

### Recolección de Datos

Para recolectar los datos se utilizaron el Formulario de Información Demográfica y la Escala de Conocimientos, Actitudes y Prácticas para COVID-19 (KAP COVID-19). La recolección de datos se llevó a cabo durante un período de ocho semanas, de mayo a julio de 2022. Se informó a los participantes sobre el estudio a través de correo electrónico y plataformas de medios sociales para fomentar la participación. Para minimizar posibles sesgos, garantizamos el anonimato en la recolección de datos para reducir el sesgo de deseabilidad social. Además, se informó a los participantes de que no había respuestas correctas o incorrectas, lo que les animó a dar respuestas sinceras. La naturaleza en línea de la recolección de datos puede haber introducido un sesgo de selección, ya que la participación requería acceso a Internet.

*Formulario de información demográfica*: este formulario fue elaborado por los investigadores para recolectar información demográfica sobre las enfermeras. El formulario consta de un total de 27 preguntas, entre las que se incluyen la edad, el sexo, el estado civil, el nivel educativo, la experiencia laboral, la duración del empleo en la clínica actual, las horas de trabajo semanales e información relacionada con el bienestar físico (sueño, ejercicio, alimentación saludable, etc.) y mental (estrés, ansiedad, etc.).

*Escala de conocimientos, actitudes y prácticas para COVID-19 (KAP COVID-19)*: Este formulario fue adaptado al turco por Kurt et al.^(12)^ a partir de la versión desarrollada por Zhong et al.^(13)^, comprende un total de 16 ítems y 5 factores. Los primeros 12 ítems de la escala están dirigidos a medir el nivel de conocimientos y constan de tres factores: Presentación Clínica (Ítems 1, 2, 3, 4), Vías de Transmisión (Ítems 5, 6, 7), y Prevención y Control (Ítems 8, 9, 10, 11, 12). La escala consta de las opciones «verdadero», «falso» y «no sabe». Se asigna 1 punto a las respuestas correctas, mientras que se asignan 0 puntos a las respuestas incorrectas o cuando se selecciona «no sabe». La puntuación total de la escala puede oscilar entre un mínimo de 0 y un máximo de 12. Las puntuaciones altas indican un alto nivel de conocimiento sobre COVID-19, mientras que las puntuaciones bajas indican un bajo nivel de conocimiento. Las actitudes hacia COVID-19 se miden mediante dos ítems relativos al consenso sobre el control definitivo de COVID-19 y la confianza en ganar la batalla contra COVID-19. Las prácticas se evalúan mediante dos ítems, en los que se pregunta si se ha acudido recientemente a un lugar concurrido y si se lleva mascarilla al salir. El valor del alfa de Cronbach fue de 0,74 para la escala general^(12)^.

### Análisis de los Datos

El análisis de los datos se realizó con el programa informático SPSS versión 25,0. Los datos se presentaron como números, porcentajes y medias. Se utilizaron pruebas paramétricas para el análisis estadístico, ya que los datos presentaban una distribución normal. Para proporcionar una estimación más precisa de los parámetros poblacionales, se calcularon intervalos de confianza (IC) del 95% para las principales dimensiones de la escala. Estos intervalos de confianza proporcionan información adicional sobre la dispersión y fiabilidad de los datos. Las puntuaciones de conocimientos, actitudes y prácticas se evaluaron con respecto a las características demográficas mediante la prueba t de muestras independientes, ANOVA unidireccional o la prueba Chi-cuadrado, según procediera. La relación entre variables se comprobó mediante el análisis de correlación de Pearson. Se utilizó un análisis de regresión lineal simple para determinar las variables predictoras del nivel de conocimientos sobre la COVID-19. La consistencia interna de la escala CAP COVID-19 se evaluó mediante el alfa de Cronbach, que resultó ser de 0,72 en el presente estudio. Un valor p < 0,05 se consideró estadísticamente significativo en todos los análisis.

### Aspectos Éticos

El estudio recibió la aprobación de la Junta de Revisión Institucional para la Investigación en Seres Humanos de la Universidad de Sinop (Aprobación nº: 2022/31, fechada el 24 de marzo de 2022). Además, se proporcionó a los participantes información sobre el propósito del estudio al principio del formulario en línea, y se les concedió acceso a los formularios después de que marcaran la Box que indicaba su voluntad de participar en el estudio. Ninguno de los autores tuvo acceso a la información de identificación personal de los encuestados que pudiera identificar a participantes individuales durante o después de la recogida de datos.

## RESULTADOS

El estudio se realizó con 354 enfermeras. La edad media de los participantes era de 33,04 ± 8,55 años (con un rango de 21 a 59), y la experiencia laboral media era de 10,83 ± 9,16 años (con un rango de 1 a 39). Se determinó que el 75,1% de los participantes eran mujeres, el 52,5% estaban casados, el 70,3% eran licenciados universitarios y el 71,2% tenían un nivel de ingresos moderado. Se comprobó que el 85,6% de los participantes no padecía ninguna enfermedad crónica, el 47,7% trabajaba en horario regular y tenía turnos de noche, y el 59,3% trabajaba entre 41 y 50 horas semanales (Tabla 1).

**Tabla 1:** Comparación de las características sociodemográficas de los participantes y las puntuaciones KAP COVID-19 – Sinop, Turquía, 2022.

	N	%	Presentación clínica	Vías de transmisión	Prevención y control	Total
**Edad**						
20–29	168	47,5	2,88 ± 0,81	1,10 ± 0,59	3,58 ± 0,85	7,56 ± 1,53
30–39	96	27,1	2,95 ± 0,91	1,14 ± 0,58	3,56 ± 0,89	7,66 ± 1,46
40–49	77	21,8	3,09 ± 0,83	1,18 ± 0,7	3,46 ± 1,10	7,74 ± 1,69
50–59	13	3,7	3,07 ± 1,03	1,23 ± 0,83	3,53 ± 0,77	7,84 ± 1,34
**Test estadístico**			F = 1,162 p = 0,324	F = 0,418 p = 0,740	F = 0,286 p = 0,836	F = 0,325 p = 0,807
**Género**						
Femenino	266	75,1	3,03 ± 0,77	1,09 ± 0,56	3,58 ± 0,84	7,71 ± 1,32
Masculino	88	24,9	2,82 ± 0,9	1,29 ± 0,76	3,62 ± 0,84	7,74 ± 1,53
**Test estadístico**			t = 2,121 p = 0,035*	t = −2,590 p = 0,010*	t = −0,347 p = 0,729	t = −0,125 p = 0,901
**Estado civil**						
Casado	186	52,5	3,02 ± 0,83	1,16 ± 0,63	3,56 ± 0,83	7,75 ± 1,24
No casado	168	47,5	2,94 ± 0,79	1,12 ± 0,61	3,62 ± 0,84	7,69 ± 1,51
**Test estadístico**			t = 0,549 p = 0,538	t = 0,644 p = 0,520	t = −0,864 p = 0,388	t = 0,050 p = 0,960
**Educación**						
Secundario vocacional en salud	38	10,7	2,34 ± 1,09	1,42 ± 0,94	3,31 ± 1,43	7,07 ± 2,69
Técnico	42	11,9	3,04 ± 0,76	0,97 ± 0,60	3,47 ± 1,01	7,50 ± 1,41
Graduado	249	70,3	3,01 ± 0,79	1,12 ± 0,55	3,59 ± 0,81	7,72 ± 1,31
Maestría o más	25	7,1	3,16 ± 0,80	1,12 ± 0,60	3,60 ± 0,70	7,88 ± 1,30
**Test estadístico**			F = 7,993 p = 0,000*	F = 3,700 p = 0,012*	F = 1,134 p = 0,335	F = 2,288 p = 0,078
**Ingresos**						
Menos que gastos	70	19,8	3,05 ± 0,79	1,21 ± 0,67	3,68 ± 0,87	7,95 ± 1,31
Igual a gastos	252	71,2	2,93 ± 0,88	1,13 ± 0,61	3,50 ± 0,95	7,58 ± 1,62
Más que gastos	32	9,0	2,87 ± 0,75	0,93 ± 0,50	3,59 ± 0,66	7,40 ± 1,18
**Test estadístico**			F = 0,699 p = 0,498	F = 2,187 p = 0,114	F = 1,066 p = 0,346	F = 2,035 p = 0,132
**Tabaquismo**						
Si	142	40,1	2,84 ± 0,93	1,23 ± 0,72	3,59 ± 1,03	7,66 ± 1,81
No	212	59,9	3,02 ± 0,79	1,07 ± 0,54	3,52 ± 0,82	7,62 ± 1,33
**Test estadístico**			t = −1,987 p = 0,048*	t = 2,405 p = 0,017*	t = 0,682 p = 0,496	t = 0,277 p = 0,782
**Uso de alcohol**						
Si	65	18,4	2,58 ± 1,05	1,21 ± 0,85	3,21 ± 1,30	7,01 ± 2,34
No	289	81,6	3,03 ± 0,77	1,11 ± 0,55	3,62 ± 0,78	7,78 ± 1,25
**Test estadístico**			t = −3,948 p = 0,000*	t = 1,141 p = 0,255	t = −3,306 p = 0,001*	t = −3690 p = 0,000*
Padecimiento de enfermedades crónicas						
Si	51	14,4	3,13 ± 0,74	1,09 ± 0,60	3,58 ± 0,87	7,82 ± 1,60
No	303	85,6	2,92 ± 0,86	1,14 ± 0,62	3,54 ± 0,92	7,61 ± 1,52
**Test estadístico**			t = 1,654 p = 0,099	t = −0,464 p = 0,643	t = 0,314 p = 0,754	t = 0,913 p = 0,362
**Experiencia de trabajo (años)**						
0–9	190	53,7	2,87 ± 0,81	1,10 ± 0,58	3,61 ± 0,83	7,59 ± 1,47
10–19	91	25,7	3,00 ± 0,86	1,21 ± 0,66	3,53 ± 0,89	7,75 ± 1,50
20–29	62	17,5	3,11 ± 0,87	1,11 ± 0,68	3,33 ± 1,172	7,56 ± 1,78
30–39	11	3,1	3,00 ± 1,18	1,18 ± 0,60	3,72 ± 0,78	7,90 ± 1,51
**Test estadístico**			F = 1,307 p = 0,272	F = 0,804 p = 0,492	F = 1,564 p = 0,197	F = 0,393 p = 0,758
**Área de trabajo**						
Emergencias	74	20,9	2,82 ± 0,88	1,16 ± 0,74	3,56 ± 0,93	7,55 ± 1,67
Cirugía	57	16,1	3,03 ± 0,80	1,05 ± 0,39	3,66 ± 0,78	7,75 ± 1,15
Medicina Interna	73	20,6	2,73 ± 0,97	1,10 ± 0,51	3,39 ± 1,12	7,24 ± 1,80
Unidad de Cuidados Intensivos	38	10,7	2,97 ± 0,67	1,07 ± 0,48	3,55 ± 0,86	7,60 ± 1,15
Otros (Policlínicas, etc.)	112	31,6	3,13 ± 0,79	1,19 ± 0,73	3,58 ± 0,83	7,91 ± 1,51
**Test estadístico**			F = 3,021 p = 0,018*	F = 0,659 p = 0,621	F = 0,770 p = 0,545	F = 3,227 p = 0,046*
**Horario de trabajo**						
Guardias	102	28,8	2,68 ± 0,91	1,01 ± 0,54	3,37 ± 1,12	7,07 ± 1,90
Sólo diurno	83	23,4	3,12 ± 0,86	1,16 ± 0,64	3,65 ± 0,73	7,93 ± 1,05
Días ± Deberes	169	47,7	3,03 ± 0,77	1,18 ± 0,65	3,60 ± 0,84	7,83 ± 1,40
**Test estadístico**			F = 7,641 p = 0,001*	F = 3,527 P = 0,044*	F = 2,785 p = 0,063	F = 10,202 p = 0,000*
**Horas de trabajo semanal**						
40 hrs. o menos	65	18,4	2,93 ± 1,02	1,07 ± 0,62	3,38 ± 1,08	7,40 ± 1,87
41–50 hrs.	210	59,3	2,89 ± 0,83	1,13 ± 0,62	3,58 ± 0,84	7,61 ± 1,47
51 hrs o más	79	22,3	3,12 ± 0,72	1,17 ± 0,63	3,59 ± 0,94	7,89 ± 1,38
**Test estadístico**			F = 2,136 p = 0,120	F = 0,463 p = 0,630	F = 1,310 p = 0,271	F = 1,933 p = 0,146

*El valor p < 0,05 se considera estadísticamente significativo.

La puntuación media de Conocimientos COVID-19 de los participantes fue de 7,64 ± 1,54 (IC del 95%: 7,48 – 7,80). La puntuación media de las presentaciones clínicas fue de 2,95 ± 0,85 (IC del 95%: 2,86 – 3,04). La puntuación media de las vías de transmisión fue de 1,14 ± 0,62 (IC del 95%: 1,07 – 1,20). La puntuación media de prevención y control fue de 3,55 ± 0,91 (IC 95%: 3,45 – 3,65). En la Tabla 1 se presentan las puntuaciones medias de la escala CAP COVID-19 y sus subdimensiones según las características sociodemográficas de los participantes. Se encontraron diferencias estadísticamente significativas en las puntuaciones de la subdimensión de presentaciones clínicas con respecto al sexo, nivel educativo, consumo de tabaco y alcohol, departamento y horario de trabajo (p < 0,05). Se observaron puntuaciones medias estadísticamente significativas más altas en las puntuaciones de las subdimensiones de las presentaciones clínicas entre los participantes que eran mujeres, tenían títulos de posgrado, no eran fumadores ni bebedores, trabajaban exclusivamente en turnos diurnos y estaban empleados en otras unidades como ambulatorios y unidades quirúrgicas. Se observaron diferencias estadísticamente significativas en las puntuaciones de las subdimensiones de las vías de transmisión con respecto al sexo, el nivel de estudios, el hábito de fumar y el horario de trabajo (p < 0,05). Se determinó que las mujeres enfermeras, las enfermeras con un título asociado, los no fumadores y las enfermeras que trabajaban por turnos tenían puntuaciones significativamente más bajas en las vías de transmisión. Se encontró una diferencia estadísticamente significativa en las puntuaciones de la subdimensión de prevención y control con respecto al consumo de alcohol, teniendo los no bebedores puntuaciones medias significativamente más altas (p < 0,05). Se encontraron diferencias estadísticamente significativas en las puntuaciones de Conocimientos COVID-19 con respecto al consumo de alcohol, el departamento y el horario de trabajo (p < 0,05). Los no bebedores, los que trabajaban exclusivamente en turnos de día, así como los que trabajaban en otras unidades como ambulatorios y unidades quirúrgicas, obtuvieron puntuaciones significativamente más altas en Conocimientos COVID-19 (Tabla 1).

Las características de los participantes en relación con el COVID-19 se presentan en la Tabla 2. Se determinó que el 60,7% de los participantes había estado infectado por COVID-19, el 37,9% tenía un familiar infectado y el 79,4% había atendido a un paciente con COVID-19. No hubo diferencias estadísticamente significativas en las puntuaciones de Conocimiento de COVID-19 entre los participantes, independientemente de si ellos mismos habían sido infectados, si sus familiares habían sido infectados o si habían proporcionado cuidados a un paciente infectado (p > 0,05). Se encontraron diferencias estadísticamente significativas en las puntuaciones de la subdimensión de presentación clínica en relación con el estado de infección de los familiares (p < 0,05). Del mismo modo, se encontraron diferencias estadísticamente significativas en las puntuaciones de las subdimensiones de prevención y control y en las puntuaciones de Conocimientos COVID-19 con respecto al contacto con pacientes infectados (p < 0,05). Se determinó que los participantes que rara vez o nunca tuvieron contacto con pacientes infectados presentaron puntuaciones significativamente más altas en Conocimientos COVID-19 y en las subdimensiones de prevención y control (Tabla 2).

**Tabla 2:** Comparación de las puntuaciones COVID-19 de CAP con respecto a otras características – Sinop, Turquía, 2022.

	N	%	Presentación clínica	Vías de transmisión	Prevención y control	Total
**Infección COVID-19**						
Si	215	60,7	3,01 ± 0,82	1,09 ± 0,56	3,58 ± 0,86	7,69 ± 1,43
No	139	39,3	2,85 ± 0,89	1,19 ± 0,70	3,50 ± 1,00	7,55 ± 1,68
**Test estadístico**			t = 1,754 p = 0,080	t = −1,362 p = 0,175	t = 0,753 p = 0,452	t = 0,857 p = 0,392
**Infección de un integrante de la familia**						
Si	134	37,9	2,88 ± 0,87	1,14 ± 0,54	3,62 ± 0,86	7,65 ± 1,48
No	220	62,1	3,00 ± 0,83	1,12 ± 0,67	3,50 ± 0,94	7,63 ± 1,57
**Test estadístico**			t = −1,277 p = 0,202	t = 0,321 p = 0,748	t = 1,217 p = 0,225	t = 0,147 p = 0,883
**Infección de un pariente**						
Si	117	33,1	2,78 ± 0,80	1,10 ± 0,57	3,53 ± 0,93	7,42 ± 1,51
No	237	66,9	3,03 ± 0,86	1,15 ± 0,64	3,55 ± 0,91	7,74 ± 1,54
**Test estadístico**			t = −2,631 p = 0,009*	t = −0,699 p = 0,485	t = −0,178 p = 0,859	t = −1,842 p = 0,066
**Cuidó de un paciente COVID-19**						
Si	281	79,4	2,96 ± 0,89	1,12 ± 0,61	3,54 ± 0,93	7,62 ± 1,57
No	73	20,6	2,93 ± 0,69	1,17 ± 0,67	3,57 ± 0,84	7,68 ± 1,42
**Test estadístico**			t = −0,699 p = 0,485	t = −0,699 p = 0,485	t = −0,699 p = 0,485	t = −0,699 p = 0,485
**Contacto con un paciente COVID-19**						
Siempre	129	36,4	3,00 ± 0,83	1,18 ± 0,69	3,64 ± 0,83	7,83 ± 1,44
A veces	155	43,8	2,87 ± 0,92	1,07 ± 0,55	3,40 ± 1,05	7,34 ± 1,67
Raramente/nunca	70	19,8	3,04 ± 0,71	1,17 ± 0,63	3,71 ± 0,66	7,92 ± 1,28
**Test estadístico**			F = 1,371 p = 0,255	F = 1,212 p = 0,299	F = 3,920 p = 0,021*	F = 5,184 p = 0,006*

*El valor p < 0,05 se considera estadísticamente significativo.

En la Tabla 3 se muestra la relación entre los niveles de salud emocional y física percibidos por los participantes durante la pandemia y las puntuaciones COVID-19 de CAP. Se encontró una correlación positiva débil y estadísticamente significativa entre las puntuaciones de Conocimiento de COVID-19 y el sentirse ansioso, estresado, deprimido y feliz durante la pandemia (p < 0,05). A medida que aumenta el nivel de conocimientos sobre COVID-19, también aumenta el nivel de sentirse ansioso, estresado, deprimido y feliz durante la pandemia. Se observó una correlación positiva débil y estadísticamente significativa entre la puntuación de las presentaciones clínicas y el sentimiento de ansiedad, estrés y felicidad durante la pandemia. Por el contrario, se observó una correlación negativa débil y estadísticamente significativa entre la puntuación de las presentaciones clínicas y el nivel de alimentación saludable. A medida que aumenta la puntuación de las presentaciones clínicas, aumenta el nivel de sentirse ansioso, estresado y deprimido durante la pandemia y disminuye el nivel de alimentación sana. Hubo una correlación positiva débil y estadísticamente significativa entre las puntuaciones de las vías de transmisión y el nivel de fatiga. A medida que aumenta la puntuación de las vías de transmisión, también aumenta el nivel de fatiga. Se encontró una correlación positiva débil y estadísticamente significativa entre las puntuaciones de prevención y control y el hecho de sentirse feliz durante la pandemia. A medida que aumenta la puntuación en prevención y control, también aumenta el nivel de sentirse feliz durante la pandemia (Tabla 3).

**Tabla 3:** Relación entre la salud emocional y física percibida por los participantes durante la pandemia y las puntuaciones COVID-19 de CAP – Sinop, Turquía, 2022.

	M ± DE	Min–Max	Presentación clínica	Vías de transmisión	Prevención y control	Total
Satisfacción con la vida laboral	5,04 ± 2,32	0–10	0,052	–0,048	0,038	0,68
Sentir ansiedad durante la pandemia	7,22 ± 2,56	0–10	0,117*	0,084	0,006	0,115*
Sentirse estresado durante la pandemia	7,23 ± 2,55	0–10	0,152**	0,067	0,032	0,159**
Sensación de depresión durante la pandemia	6,75 ± 2,63	0–10	0,090	0,078	0,052	0,135*
Nivel de felicidad durante la pandemia	6,71 ± 2,38	0–10	0,182**	0,068	0,126*	0,208**
Nivel de sueño	5,08 ± 2,39	0–10	–0,062	–0,007	0,044	–0,007
Nivel de cansancio	7,36 ± 2,13	0–10	–0,003	0,144**	0,052	0,086
Nivel de actividad física	6,58 ± 2,31	0–10	–0,063	0,024	0,073	0,028
Nivel de alimentación saludable	6,23 ± 2,08	0–10	–0,128*	0,025	0,004	–0,085

Se utilizó un análisis de regresión lineal simple para determinar las variables predictoras del nivel de conocimientos sobre el COVID-19 (Tabla 4). El análisis reveló que el nivel de sentirse feliz durante la pandemia, trabajar sólo en turnos diurnos, no consumir alcohol y no tener contacto con un paciente infectado, explicaban 10% de la varianza en el nivel de conocimiento de los participantes sobre COVID-19 (p < 0,001).

**Tabla 4:** Resultados del análisis de regresión lineal predictivo del nivel de conocimientos sobre COVID-19 – Sinop, Turquía, 2022.

Variables	B	S.H.	β	t	p	VIF
Constante	6,295	,328		19,183	,000	1,009
Nivel de felicidad durante la pandemia	,144	,033	,224	4,386	,000	1,023
Horario de trabajo	,240	,186	,066	1,286	,199	1,015
Uso de alcohol	,695	,203	,175	3,419	,001	1,018
Contacto con paciente infectado	−,307	,198	−,080	−1,553	,121	1,009
R = ,316 R^2^ = ,100 F = 9,662 p < 0,001						

Cuando se analizó la subdimensión de actitud de la escala, se observó que el 61% (216) de los participantes respondieron «De acuerdo» a la pregunta «¿Piensa que el COVID-19 será finalmente controlado con éxito?», y el 72,9% (258) de los participantes respondieron «Sí» a la pregunta «¿Tiene confianza en que Turquía puede ganar la batalla al virus COVID-19?». Cuando se analizó la subdimensión de prácticas de la escala, se observó que el 81,1% (287) de los participantes respondieron «Sí» a la pregunta «¿Visito recientemente algún lugar concurrido?», y el 74,6% (264) respondieron «No» a la pregunta «¿Usa mascarilla al salir de casa en los últimos días?» (Figura 1).

**Figura 1:** Actitudes y prácticas en relación al COVID-19.

## DISCUSIÓN

La prevención es la única solución para una enfermedad que no dispone de tratamiento curativo. Mientras la mayoría de la población no esté vacunada y el país alcance el nivel de inmunidad de rebaño requerido, las medidas preventivas dependen en gran medida del CAP de la población afectada. Los conocimientos y el comportamiento del personal sanitario son cruciales para prevenir la propagación de la infección. Si se descuidan, los trabajadores sanitarios no sólo corren el riesgo de convertirse ellos mismos en vectores potenciales, sino también de poner en peligro sus propias vidas^(14)^. Este estudio se llevó a cabo durante el período tardío de la COVID-19 en Turquía para investigar la CAP en relación con la gestión de la COVID-19 entre las enfermeras en el lugar de trabajo y en su vida personal. Hasta donde sabemos, este estudio es la primera evaluación exhaustiva de los conocimientos, actitudes y prácticas entre los trabajadores sanitarios de primera línea, en particular las enfermeras, en relación con la fase tardía de la COVID-19 en Turquía. En esta población predominantemente femenina y bien educada, la tasa de respuesta correcta en el cuestionario de conocimientos fue del 63,67%, con una puntuación media de conocimientos de 7,64 ± 1,54.

Estos resultados indican que la mayoría de los encuestados tenían un conocimiento moderado de la COVID-19. La puntuación de conocimientos obtenida entre los trabajadores sanitarios en el presente estudio fue inferior en comparación con otros estudios internacionales realizados en Nigeria (92%), China (89%) y Arabia Saudí (84%)^(11,15,16)^. En otros estudios realizados en Turquía se observó que el nivel de conocimientos de los trabajadores sanitarios sobre la COVID-19 era del 92% y el 94%, respectivamente, que eran superiores en comparación con el presente estudio^(17,18)^. Del mismo modo, Ejeh et al.^(19)^ observaron que el 88% de los profesionales sanitarios respondían correctamente a las preguntas, con una puntuación media de conocimientos de 7,1. Estos estudios se llevaron a cabo durante las primeras fases de la pandemia, cuando la información disponible era limitada. En cambio, el presente estudio se realizó durante una fase posterior, cuando se disponía de abundante información y las experiencias personales también influían en las opiniones. Esta diferencia puede haber dado lugar a diferencias en los resultados. También existen en la bibliografía resultados coherentes con el presente estudio^(20,21,22,23)^. Además de esta información, el presente estudio reveló que la mayoría de las enfermeras tenían conocimientos suficientes sobre los síntomas y signos clínicos de la enfermedad (73,75%), así como sobre sus medidas de prevención y control (71%). Sin embargo, sus conocimientos sobre los modos de transmisión (37,67%) eran insuficientes. Estos resultados coinciden con los de estudios realizados en Vietnam y la India, que sugieren que el personal sanitario tiene conocimientos suficientes sobre la transmisión y los síntomas clínicos de la COVID-19^(21,24)^. De forma similar, Bangani et al.^(22)^ hallaron puntuaciones moderadamente buenas (78%), bajas (55%) y moderadas (65%) en los conocimientos del personal de enfermería de cuidados intensivos sobre la COVID-19, puntuaciones de actitud y puntuaciones de práctica. En este estudio, realizado durante la fase intermedia de la COVID-19, se observó que las puntuaciones de actitud especialmente bajas estaban influidas por la escasa formación en prevención y control de infecciones, el tiempo insuficiente para su aplicación y la falta de equipos de protección individual. Las bajas puntuaciones en el presente estudio pueden deberse a la aparición de una plétora de nueva información relativa a la propagación de la enfermedad y las vías de transmisión durante el periodo de estudio, junto con una formación inadecuada proporcionada a las enfermeras sobre esta nueva información. Además, la incapacidad de las enfermeras para mantenerse al día de la información debido a sus exigentes horarios de trabajo puede haber contribuido a esta situación. Los análisis realizados en el presente estudio revelaron que, en general, las enfermeras con un nivel de estudios de licenciatura o superior, que no consumían alcohol ni tabaco y que trabajaban sólo en turnos de día en unidades ambulatorias, tendían a tener puntuaciones de conocimientos más altas. Tener un nivel de estudios superior es uno de los factores que contribuyen a tener más experiencia debido al mayor acceso a la información y a las oportunidades de adquisición de conocimientos. En el presente estudio, se observó que las enfermeras con una licenciatura o un nivel educativo superior tenían niveles más altos de conocimientos sobre la COVID-19 en comparación con las que tenían un nivel educativo inferior, lo que concuerda con un estudio anterior realizado en Turquía^(17)^. En un estudio similar realizado en Arabia Saudí, Almohammed et al.^(20)^ informaron de que el sexo femenino y el nivel educativo superior afectaban positivamente a las puntuaciones de conocimiento. Este hallazgo está respaldado por estudios internacionales realizados en Yemen, Etiopía y Nigeria^(15,25,26)^. Una posible razón para ello podría ser que los profesionales sanitarios con un mayor nivel educativo pueden tener mejores oportunidades de acceder a plataformas locales e internacionales de conocimiento, investigación y formación que sus colegas^(26)^. También hay estudios en los que, contrariamente a nuestros hallazgos, no se encontraron diferencias significativas entre las características sociodemográficas y los niveles de conocimiento^(16,19)^.

En el presente estudio, investigamos la relación entre el estado de salud emocional y físico autodeclarado por los participantes y su nivel de conocimientos sobre la COVID-19. Se descubrió que, durante la pandemia, los niveles de ansiedad, estrés y depresión también aumentaban a medida que aumentaba el nivel de conocimientos. Como se indicó en un estudio anterior, teniendo en cuenta el grave impacto de la pandemia en la sociedad, se espera que haya una presencia general de miedo y ansiedad en la comunidad; esto también puede afectar a la salud mental de las personas y a los comportamientos sociales^(27)^. Alrubaiee et al.^(25)^ hallaron una relación positiva significativa entre el nivel de conocimientos y la ansiedad, similar a la del presente estudio. Se observó que los niveles de ansiedad entre los trabajadores sanitarios podrían atribuirse a la preocupación por infectarse, sobre todo teniendo en cuenta la naturaleza incontrolable del brote y factores como la escasez de instalaciones sanitarias y de equipos de protección personal. En el presente estudio, encontramos una correlación positiva entre el nivel de conocimientos sobre los síntomas clínicos de la COVID-19 y la sensación de ansiedad y estrés durante el periodo pandémico. Por el contrario, se encontró una correlación negativa entre el nivel de alimentación saludable y estos sentimientos. La similitud entre los síntomas y signos de la enfermedad y los síntomas clásicos de las infecciones de las vías respiratorias superiores, junto con los síntomas específicos de la enfermedad, pueden haber hecho que los participantes se preocuparan y experimentaran sentimientos emocionales negativos cuando ellos o sus seres queridos presentaban estos síntomas. La disminución de la alimentación saludable puede atribuirse al deterioro de los hábitos alimentarios como consecuencia de la ansiedad. Contrariamente a los demás resultados, se observó que los niveles de felicidad de algunos participantes aumentaban a medida que aumentaba su nivel de conocimientos. Este fenómeno puede deberse a un aumento de la confianza en sí mismos de los trabajadores a medida que aumenta su nivel de conocimientos, lo que les lleva a sentirse más seguros frente a la transmisión del virus. Se observó una correlación positiva significativa entre el nivel de conocimientos sobre las vías de transmisión y la fatiga. El conocimiento de las vías de transmisión del virus podría llevar a las enfermeras a realizar más esfuerzos para prevenir posibles transmisiones, lo que aumentaría su carga de trabajo y provocaría una mayor fatiga.

En el presente estudio, se llevó a cabo un análisis de regresión para determinar los factores predictivos del nivel de conocimientos sobre la COVID-19. Los resultados mostraron que sentirse feliz durante la pandemia, trabajar en turnos de día, abstenerse de consumir alcohol y evitar el contacto con pacientes infectados explicaban el 10% de la varianza en el nivel de conocimientos de los participantes (p < 0,001). Estos resultados son importantes, ya que hay pocos estudios que examinen el CAP entre las enfermeras con respecto a diversas características. Además, la identificación de los factores demográficos asociados con el CAP podría ser beneficiosa para los responsables de las políticas de salud pública y los profesionales sanitarios a la hora de reconocer las poblaciones diana para la prevención del COVID-19 y las iniciativas de educación sanitaria.

La mayoría de las enfermeras mostraron una actitud optimista ante la pandemia del COVID-19: el 61% creía que el COVID-19 se controlaría con éxito, y el 72,9% creía que Turquía podría ganar la batalla contra el virus. En un estudio en el que se investigaron los conocimientos, las conductas preventivas y la percepción del riesgo del personal sanitario en relación con la pandemia de COVID-19 en Turquía, se informó de que la gran mayoría de los participantes (84%) creía que el brote se controlaría^(17)^. Estos índices, a pesar de haberse realizado en las primeras fases de la pandemia, son más optimistas en comparación con el presente estudio. Los resultados obtenidos en el presente estudio pueden atribuirse al hecho de que la pandemia lleva aproximadamente tres años en curso y aún no se ha controlado por completo. En el momento en que se realizó este estudio, se habían levantado las restricciones preventivas para COVID-19 aplicadas en Turquía. Esto puede haber dado lugar a las prácticas menos estrictas observadas: La gran mayoría de los participantes declaró acudir a lugares concurridos (81,1%) y no llevar mascarilla al salir de casa (74,6%).

Este hallazgo concuerda con estudios anteriores que han demostrado un descenso en la adherencia a las medidas preventivas a medida que disminuía la percepción pública del riesgo. Petherick et al.^(28)^ descubrieron que la adherencia a las conductas de protección disminuía globalmente a medida que se relajaban las restricciones y aparecía la fatiga pandémica. Del mismo modo, Seyd y Bu^(29)^ destacaron que el cumplimiento público de las medidas COVID-19 estaba fuertemente influenciado por la percepción del riesgo y la confianza en las instituciones gubernamentales, y que los niveles de cumplimiento disminuían a medida que disminuía la preocupación por el virus. Estos resultados sugieren que la adhesión a largo plazo a las medidas de protección está determinada de forma significativa tanto por la confianza institucional como por los niveles de amenaza percibidos. En otro estudio realizado durante las primeras fases de la pandemia, Arslanca et al.^(17)^ informaron de que los principales comportamientos preventivos fuera del trabajo eran el uso de mascarillas (96%) y evitar los lugares concurridos (91,63%). Aunque nuestro estudio se llevó a cabo durante un periodo en el que se habían abolido las restricciones destinadas a controlar la pandemia, el hecho de que casi el 20–30% de los participantes siguieran adhiriéndose voluntariamente a las medidas preventivas es indicativo de su continua preocupación. De forma similar a nuestro estudio, en un estudio realizado por Juttla et al.^(30)^ durante las fases medias de la pandemia en Kenia, se informó de que la mayoría de los trabajadores sanitarios tenían una actitud negativa hacia el COVID-19, a pesar de tener un alto nivel de prácticas preventivas. Se observó que el 66,3% de ellos evitaba a los pacientes con síntomas y signos de COVID-19. Los autores destacaron que esto indicaba la falta de confianza de los trabajadores sanitarios en la eficacia de los protocolos preventivos, independientemente de su adhesión a los mismos. El clima organizativo puede influir significativamente en los conocimientos, las actitudes y las prácticas de las enfermeras al influir en el acceso a la formación, la disponibilidad de medidas de protección y las políticas institucionales. Nuestra hipótesis es que un clima organizativo favorable, caracterizado por una formación continua y unas políticas claras de control de la infección, facilita un mayor cumplimiento de las medidas preventivas por parte del personal de enfermería.

El presente estudio tiene ciertas limitaciones. Una de ellas es que el diseño transversal de la investigación puede no explicar suficientemente la relación causa-efecto. Además, el estudio se realizó en un único centro y no se tuvieron en cuenta las experiencias de los trabajadores sanitarios del sector privado. Por lo tanto, los resultados no pueden generalizarse a toda la población. Otra limitación es que este estudio se realizó mediante una encuesta en línea. Las respuestas dependían de la honestidad y estaban influidas por la capacidad de recordar; por lo tanto, pueden estar sujetas a un sesgo de recuerdo. Sin embargo, un punto fuerte notable de este estudio es que participaron todas las enfermeras que trabajaban en un gran hospital estatal de Turquía. Además, este es el primer estudio que evalúa el CAP de las enfermeras con respecto a la COVID-19 durante las últimas fases de la pandemia. Los datos obtenidos pueden servir como un valioso recurso para futuras investigaciones y esfuerzos educativos.

## CONCLUSIÓN

Los resultados obtenidos en el presente estudio revelaron importantes factores relacionados con el CAP entre las enfermeras que sobrevivieron a la infección por COVID-19. Estos resultados suponen una importante contribución a la literatura en cuanto a que reflejan los hallazgos de la fase tardía de la pandemia, ya que el estudio se realizó en un momento en el que se habían levantado las medidas preventivas. En general, se constató que las enfermeras tenían un nivel moderado de conocimientos, una actitud positiva hacia el control de la pandemia y la capacidad de Turquía para ganar la batalla contra este virus. Sin embargo, se observó una relajación perceptible en sus prácticas relacionadas con el COVID-19, como el uso de mascarillas y evitar los lugares concurridos. Además, se observó que el sexo femenino y un mayor nivel educativo influían en el aumento de las puntuaciones de conocimientos. La identificación de los factores que influyen en las actitudes y comportamientos de los profesionales sanitarios ante el virus puede orientar el desarrollo de programas de formación y políticas específicas durante la pandemia. Estos esfuerzos pueden centrarse en la protección de los trabajadores sanitarios y ayudarles a evitar la exposición ocupacional. Los resultados también revelaron que las enfermeras que sobrevivieron al COVID-19 seguían experimentando sentimientos de ansiedad, estrés y depresión durante el periodo pandémico. Sobre la base de estos hallazgos, se puede recomendar proporcionar apoyo psicológico en el lugar de trabajo centrándose en mejorar la satisfacción laboral y mejorar la calidad de los resultados de salud entre las enfermeras haciendo hincapié en la creación de resiliencia, la capacitación y la prevención del agotamiento. Además, los hospitales deberían ofrecer programas de desarrollo profesional continuo para concienciar a todos los trabajadores sanitarios y apoyar su bienestar emocional durante la pandemia en curso. Teniendo en cuenta que los datos se recolectaron en 2022, los análisis adicionales podrían incluir un estudio de seguimiento longitudinal para evaluar cómo evolucionan con el tiempo los conocimientos, las actitudes y las prácticas de las enfermeras.

Los estudios comparativos con conjuntos de datos más recientes también podrían ayudar a determinar si el cumplimiento de las medidas preventivas de la COVID-19 ha seguido disminuyendo o mejorando después de la pandemia.

## Data Availability

Los datos que respaldan los hallazgos de este estudio están disponibles a través del autor correspondiente previa solicitud razonable.
